# 

**Published:** 1875-02-01

**Authors:** 


No. III. FEBRUARY 1, 1875. Vol. XVI.

S.G.O.L INDEXED

### THE MEDICAL EXAMINER:

A

Semi-Monthly Journal of Medical Sciences.

EDITED BY

N. S. DAVIS, M. D., and F. H. DAVIS, M. D.

*Subscription Price, Three Dollars per Annum, in advance.*
*Single Numbers, Fifteen Cents.*

CHICAGO.

PUBLISHED AT 65 E. RANDOLPH STREET.

